# Concerted Spatio-Temporal Dynamics of Imported DNA and ComE DNA Uptake Protein during Gonococcal Transformation

**DOI:** 10.1371/journal.ppat.1004043

**Published:** 2014-04-24

**Authors:** Heike Gangel, Christof Hepp, Stephanie Müller, Enno R. Oldewurtel, Finn Erik Aas, Michael Koomey, Berenike Maier

**Affiliations:** 1 Department of Physics, University of Cologne, Cologne, Germany; 2 Department of Biosciences, University of Oslo, Oslo, Norway; University of Minnesota, United States of America

## Abstract

Competence for transformation is widespread among bacterial species. In the case of Gram-negative systems, a key step to transformation is the import of DNA across the outer membrane. Although multiple factors are known to affect DNA transport, little is known about the dynamics of DNA import. Here, we characterized the spatio-temporal dynamics of DNA import into the periplasm of *Neisseria gonorrhoeae*. DNA was imported into the periplasm at random locations around the cell contour. Subsequently, it was recruited at the septum of diplococci at a time scale that increased with DNA length. We found using fluorescent DNA that the periplasm was saturable within minutes with ∼40 kbp DNA. The DNA-binding protein ComE quantitatively governed the carrying capacity of the periplasm in a gene-dosage-dependent fashion. As seen using a fluorescent-tagged derivative protein, ComE was homogeneously distributed in the periplasm in the absence of external DNA. Upon addition of external DNA, ComE was relocalized to form discrete foci colocalized with imported DNA. We conclude that the periplasm can act as a considerable reservoir for imported DNA with ComE governing the amount of DNA stored potentially for transport through the inner membrane.

## Author Summary

Bacterial transformation is the import and inheritable integration of external DNA. As such, it is believed to be a major evolutionary force. A key step is the import of DNA through the outer membrane. Here, we have characterized the spatio-temporal dynamics of DNA during import and residence in the periplasm of the Gram-negative pathogen *Neisseria gonorrhoeae*. We found that the periplasm can serve as a reservoir for imported DNA that can fill within five minutes by importing DNA from the environment. The amount of imported DNA roughly corresponds to the size of a phage genome. The periplasmic DNA-binding protein ComE is homogeneously distributed in the periplasm in the absence of extracellular DNA. It relocates rapidly to imported DNA when external DNA is added to competent gonococci. As ComE governs the carrying capacity of the periplasm, we propose that it might condense DNA, thus linking DNA uptake to its compaction. Although the import through the outer membrane was localized all around the cell contour, the major part of the imported DNA relocated to the septum at the center of diplococci. Our findings strongly support the idea that the periplasm masses DNA independently of transport through the inner membrane.

## Introduction

Natural competence for transformation is widespread among different bacterial species [1]. Transformation is thought to speed up adaptive evolution but it is also discussed in the context of genome maintenance [2] [3]. The currently available data from Gram-positive species strongly supports the idea of a coordinated DNA transformation machine that binds DNA at the extracellular side, powers translocation of DNA through the cell envelope and hands the DNA over to the recombination machine at the intracellular side [4] [5]. With the only known exception of *Helicobacter pylori*, all characterized naturally competent species are associated with the type IV pilus (T4P) system for DNA import. At the extracellular side, T4P proteins are essential for DNA binding although it is unclear whether long pilus filaments are necessary [1]. Whereas DNA binding to the competence pilus has been demonstrated in *Streptococcus pneumoniae* [6], *Neisseria gonorrhoeae* that generate non-retractile T4P show strongly impaired binding efficiency [7]. In the following, the nomenclature of *N. gonorrhoeae* will be used to describe the proteins required for transformation. The major pilin subunit is essential for binding and import of DNA [8] [7] but replacing the gonococcal pilin PilE by the major subunit of *Pseudomonas aeruginosa* or of *Fracisella tularensis* supports DNA import and transformation as well [9], [10]. The transformation rate is modulated by the relative levels of the minor pilins ComP. PilV acts antagonistically at the level of DNA binding with ComP increasing transformability in a dose-dependent fashion and PilV decreasing it [7], [11]. PilV appears to exert its inhibitory effects by competing with ComP for access to the Tfp assembly machinery [11]. The presence of a 12 bp DNA Uptake Sequence (DUS) strongly enhances the probability for DNA-import by *N. gonorrhoeae* [12] [3]. ComP binds DNA in a sequence-specific manner, selecting for DNA containing the DUS [13] [7]. The outer membrane channel formed by PilQ is essential for T4P extrusion and DNA import into a DNase-resistant state and moreover shows DNA-binding potential [14], [15]. In the periplasm, three components are linked to transformation. The DNA-binding protein ComE has four identical gene-copies on the gonococcal genome [16]. Gradual deletion of these copies leads to gradual decrease in transformation rate by decreasing the probability for DNA import [16]. The DNA-binding peptidoglycan-linked lipoprotein ComL and the lipoprotein tetrapac (Tpc) which is associated with separation of dividing diplococci are not essential for DNA uptake but for transformation [17] [18]. ComA proteins form the channel through which DNA is transported from the periplasm to the cytoplasm [19]. In the Gram-positive species *Bacillus subtilis* it has been shown that incoming ssDNA is immediately coated by single-strand binding proteins [20]. Single strand binding proteins have been proposed to generate a reservoir of ssDNA in the cytoplasm and to direct the DNA to homologous recombination in *B. subtilis* and *Streptococcus pneumoniae* [21] [4]. For *N. gonorrhoeae* there is evidence that ssDNA forms transiently in the periplasm [22].

DNA import has been characterized at the single molecule level for *B. subtilis* and *H. pylori* [23] [24] [25] [26]. DNA uptake in *B. subtilis* proceeds at a rate of 80 bp/s at low external forces. Application of force using laser tweezers showed that the import was irreversible for forces up to 50 pN. The speed of DNA import was considerably larger in *H. pylori* with 1.3 kbp/s at low force. Application of 23 pN or more triggered extrusion of the previously imported DNA here, demonstrating the import into the periplasm is reversible. Transport through the outer and the inner membrane of *H. pylori* are not coupled in time. *H. pylori* imports fluorescently labeled Cy3-DNA into the periplasm at a somewhat decreased rate relative to unlabelled DNA [24]. Like the case of the ComEC channel based system in *B. subtilis*, the *H. pylori* ComEC-based system does not support transport of Cy3-DNA into the cytoplasm [24], suggesting that the processes for inner membrane transport are analogous between these competent species [27].

In rod-like *B. subtilis*, DNA uptake complexes form at the poles or at the growing septa [28] [20]. They are stable even when the cell wall is disrupted [29]. Accordingly, DNA uptake proceeds preferentially from the cell poles. The homologue of gonococcal ComE, ComEA, distributes homogeneously around the cell contour and is thought to enhance initial DNA binding to the cell surface [30] [31]. *H. pylori* accumulates imported Cy3-DNA primarily at the cell poles and at the septa [24]. Very recently, it has been shown that competent Gram-positive *S. pneumoniae* recruits Cy3-DNA and ComEA at midcell location [32]. It is unknown, however, where DNA uptake occurs in Gram-negative cocci.

Here, we visualized the spatio-temporal dynamics of DNA during import from the environment into the periplasm and within the periplasm of *N. gonorrhoeae*. We validated the approach of using fluorescent Cy3-DNA by characterizing a number of mutant backgrounds that had been shown to be impaired for DNA uptake. We found that DNA import through the outer membrane occurred all around the cell contour. The periplasm was saturable with DNA and held a considerable amount of Cy3-DNA. With short fragments, saturation occurred on a time scale of minutes. The periplasmic DNA-binding protein ComE strongly affected the carrying capacity of the periplasm, suggesting a role in either removing DNA from the uptake machine or in compacting DNA.

## Results

### *N. gonorrhoeae* imports Cy3-DNA into a DNase-resistant state

To investigate whether Cy3-DNA could be used to study DNA uptake in gonococci, we incubated gonococci with a 3 kbp fragment of Cy3-DNA containing one DNA uptake sequence (DUS) for 30 min and subsequently treated them with DNase. DNA was labeled randomly along its entire contour. Throughout this work, we used a *recA_ind_* background without induction to prevent the cells from antigenic variation of *pilE*. The *recA_ind_* strain will be labeled wt in the following, since it shows wt behavior in terms of DNA import through the cell envelope. Wt cells were associated with fluorescent foci that were clearly distinguishable from the fluorescence background (Fig. S1a), indicating that Cy3-DNA was imported into a DNase-resistant state.

**Figure 1:**
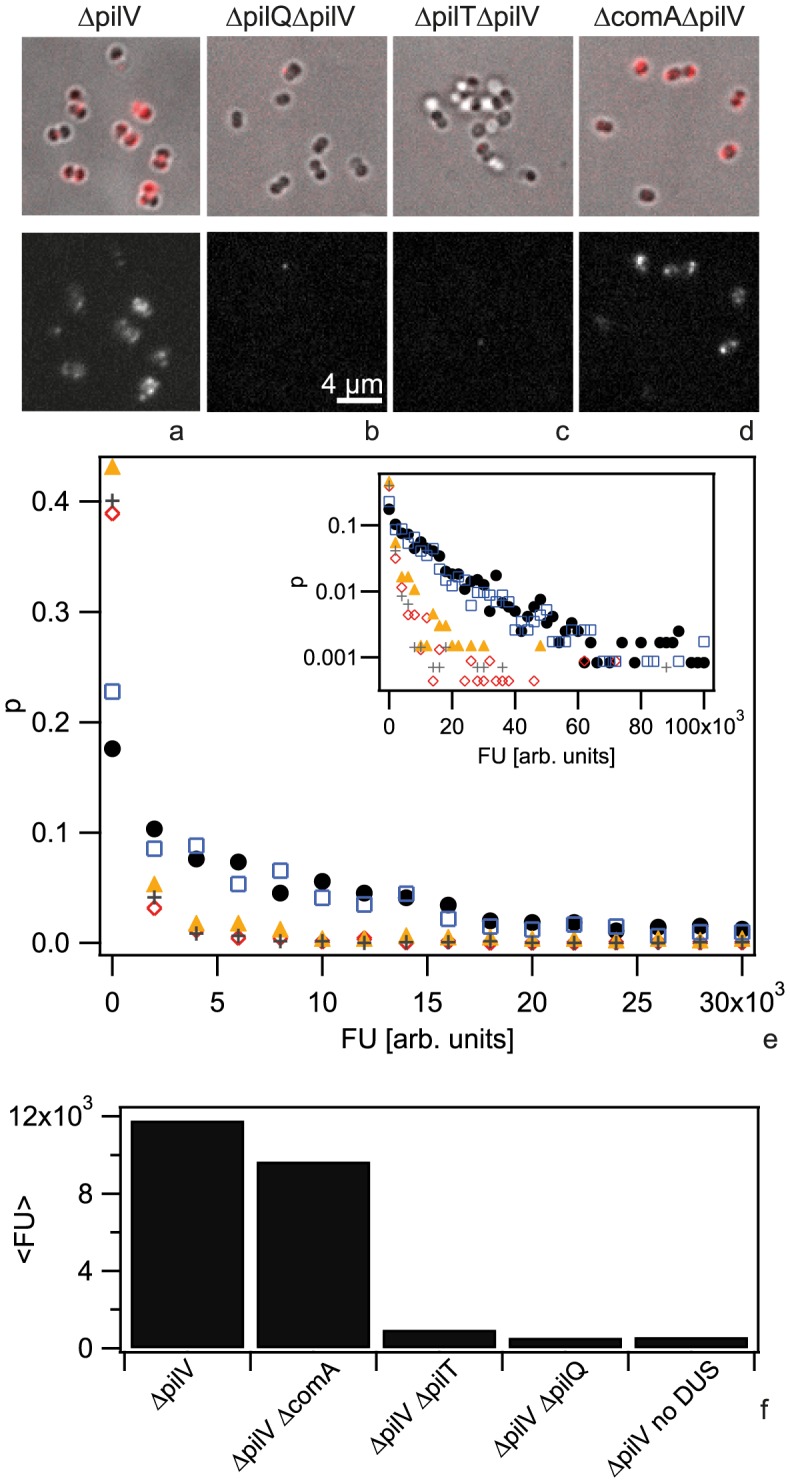
Cy3-DNA is imported into the periplasm. Gonococci were incubated with Cy3-DNA for 30 min and subsequently treated with DNase. a,b,c,d) Upper line: merged images of brightfield and fluorescence, lower line: fluorescence images. a) *ΔpilV*, b) *ΔpilQ ΔpilV*, c) *ΔpilT ΔpilV*, d) *ΔcomA ΔpilV*. e) Probability distribution of single cell fluorescence with *ΔpilV* (black), *ΔcomA ΔpilV* (blue), *ΔpilQ ΔpilV* (red), *ΔpilT ΔpilV* (orange), *ΔpilV* no DUS (grey). Inset: logarithmic representation. f) Average fluorescence intensity. (N>500 for each condition).

The minor pilins ComP and PilV have been shown to strongly affect DNA uptake at the level of DNA binding [7], [11]. We investigated the effect of these minor pilins on the uptake of Cy3-DNA. We performed the DNA uptake assay in a ComP overproducing strain (*P_pilE_comP*) and in a *pilV* deletion strain (*ΔpilV*) (Fig. 1a). As expected, both strains showed a strongly increased fluorescence signal (Fig. S1b–d), indicating that more Cy3-DNA was imported within 30 min. Since the high signal of the *ΔpilV* strain was very convenient for data analysis, we performed all following experiments in this background.

Up until now, DNA uptake by *N. gonorrhoeae* has been investigated at the population level. Therefore, it is unclear whether there is heterogeneity in this process at the level of single cells. To investigate any potential for heterogeneity, we measured the distribution of DNA uptake efficiencies of individual cells. We incubated gonococci with the 3 kbp Cy3 fragment for 30 min, treated the cells with DNase and subsequently quantified the fluorescence intensity of individual cells (Fig. S2). We found that the distribution of fluorescence intensities was very broad (Fig. 1e). A fraction of cells showed no import of DNA and this fraction was variable between different experiments. Most of the cells in our samples were diplococci. For our analysis we did not distinguish between monococci and diplococci, as the difference in the histograms was visible but small (Fig. S3).

To determine the background level of fluorescence, we repeated the experiment using a strain with a deletion in the retraction ATPase PilT (Fig. 1b) which is unable to import DNA [7]. Furthermore, we investigated a *pilQ* deletion strain that does not form the outer membrane pore, is deficient in DNA uptake, and shows reduced DNA binding (Fig. 1c). We found that both mutations strongly shifted the distribution of fluorescence intensities to lower values (Fig. 1e). Although the distributions were very broad we calculated the average fluorescence values (Fig. 1f). We found that in the *pilT pilV* background strain, the amount of DNase-resistant Cy3-DNA was reduced by a factor of 12 as compared to the *ΔpilV* strain and in the *ΔpilQΔpilV* strain the amount of DNase-resistant Cy3-DNA was reduced by a factor of 22. The strong decrease in fluorescence is consistent with previous experiments using radioactively labeled DNA although the factors are considerably lower [7], [11], suggesting that the Cy3-DNA methodology has reduced sensitivity.

Next, we investigated whether import of Cy3-DNA into a DNase-resistant state was dependent on the putative inner membrane channel formed by ComA (Fig. 1d). When repeating the DNA uptake experiment using a *comA* deletion strain (*ΔcomAΔpilV*) we found that the fluorescence distribution was not significantly different from the *ΔpilV* strain (Fig. 1e, f), indicating that the imported DNA accumulated in the periplasm. This result was analogous to what was observed previously for *H. pylori* [24].

Transformation of *N. gonorrhoeae* depends on the DNA uptake sequence (DUS). ComP has been shown to be a positive effector of sequence-specific DNA binding and that it is directly involved in binding of DUS-containing DNA [7] [13]. The DNA uptake assay with fragments lacking the DUS showed strongly reduced fluorescence (Fig. 1e, f). Thus, the uptake of Cy3-DNA was strongly enhanced by the DUS.

We conclude that Cy3-DNA is imported into the periplasm of gonococci and that DUS-related import is dependent on the outer membrane channel formed by PilQ and on the T4P retraction ATPase PilT, but not on the inner membrane channel ComA. Since these results are consistent with previous DNA uptake studies using radioactively labeled DNA, they validate our method for studying DNA uptake using *N. gonorrhoeae* Cy3-DNA. In contrast to the classic method using radioactively labeled DNA, our approach enables us to study DNA uptake at the single cell level and thus reveals strong cell-to-cell variability in terms of the total amount of DNA imported after 30 min.

### The periplasm of *N. gonorrhoeae* can retain ample amounts of imported DNA

**Figure 2:**
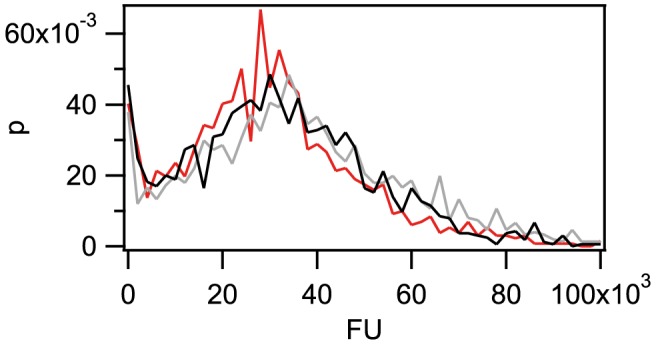
The amount of imported Cy3-DNA after 1 h is independent of fragment length. Probability distribution of the total fluorescence intensity of individual cells with a length of 300(red), 1 kbp (grey), 10 kbp (black). (N>1300 for each condition).

We investigated whether the periplasm acts as a reservoir for imported DNA. *ΔpilV* cells were incubated with Cy3-DNA for 1 h, treated with DNase and subsequently single cell fluorescence was measured. The total fluorescence intensity per cell did not vary when incubated with Cy3-DNA with fragment lengths of 0.3 kbp, 1 kbp, and 10 kbp at equimolar concentrations (Fig. 2), indicating that the total amount of imported DNA was independent of fragment length. To convert fluorescence intensity into amount of DNA, we quantified the fluorescence intensity of individual 6 kbp fragments (Fig. S4) [33] and compared them to the total fluorescence of individual cells that were incubated with Cy3-DNA from the same labeling reaction for 1 h. We found that the periplasm contained ∼40 kbp of Cy3-DNA. We note that this value might be biased slightly by binding of proteins to the DNA in the periplasm [34]. Furthermore, the Cy3-labeling efficiency is somewhat variable and therefore the fluorescence intensity cannot be directly compared to other experiments using different Cy3-DNA stocks.

**Figure 3:**
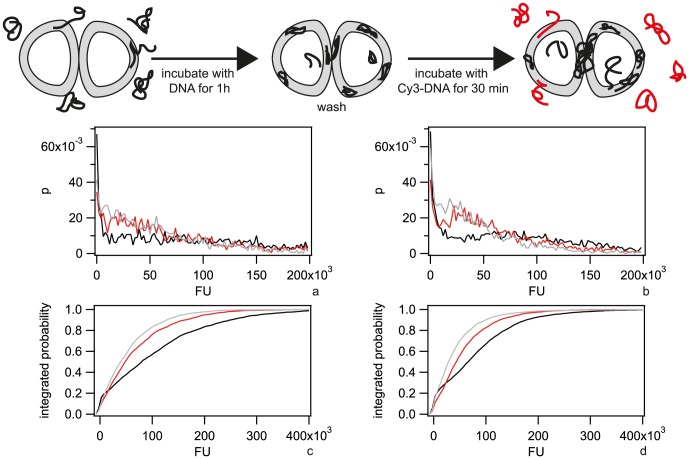
Pre-incubation with DNA inhibits uptake of Cy3-DNA. a) Probability distribution of the total fluorescence intensity of individual *ΔpilV* cells after 30 min incubation with Cy3-DNA following incubation with no DNA (black), gDNA (red), 3 kbp fragments (grey). b) Probability distribution of the total fluorescence intensity of individual *ΔcomA ΔpilV* after 30 min incubation with Cy3-DNA following incubation with no DNA (black), gDNA (red), 3 kbp fragments (grey). c) and d) are the cumulative histograms of a) and b), respectively. (N>1000 for each condition).

Next, we addressed the question whether the periplasm can be saturated with taken up DNA. To this end, we incubated gonococci for 1 h with unlabeled genomic DNA (gDNA) or 3 kbp fragments containing DUS, washed the cells, subsequently incubated them with 3 kbp Cy3-DNA for 30 min and finally treated them with DNase. We found that the distribution of fluorescence intensities was clearly shifted towards lower values when cells were pre-incubated with DNA (Fig. 3a), indicating that ample amounts of DNA remain within the periplasm during at least 30 min. Deletion of the inner membrane channel ComA did not affect saturation strongly (Fig. 3b). For averaging over the fluctuations in the histogram, we integrated over the distribution of single cell fluorescence (Fig. 3 c,d). This cumulative histogram shows the fraction of cells with fluorescence intensity (FU) up to a given value. Furthermore, we examined the stability and integrity of the imported DNA. Cells were incubated with unlabeled 10 kbp fragments for 1 h, subsequently treated with DNase, and further incubated for various periods of time. Exploiting a protocol recently developed for *Vibrio cholerae* [35], we used duplex PCR with primer pairs against the newly imported 10 kbp fragment and against gDNA of gonococci. We found that the 3 kbp DNA fragments amplified from the imported DNA were clearly detectable even after 60 min after DNase treatment in *ΔpilV* and *ΔpilV ΔcomA* backgrounds (Fig. S5). Using wt, the 3 kbp DNA fragments were still detectable after 30 min. Since the signal was lower for wt cells, we did not attempt to amplify DNA at later time points.

DNA turnover in the periplasm may occur as a consequence of dilution due to cell division, degradation, or export. To address turnover, we incubated cells with Cy3-DNA for 1 h, washed them, and subsequently incubated them for a period of 1 h either in the presence or in the absence of unlabeled DNA. We found that the distribution of fluorescence intensities shifted towards lower values independent of ComA after incubation (Fig. S6 a, b), indicating that turnover occurred and was independent of transport through the inner membrane.

Together, these data show that DNA import through the outer membrane occurs independently of inner membrane transport and that large amounts of DNA can be amassed in the periplasm over a time scale of hours.

### Kinetics of Cy3-DNA import into the periplasm

**Figure 4:**
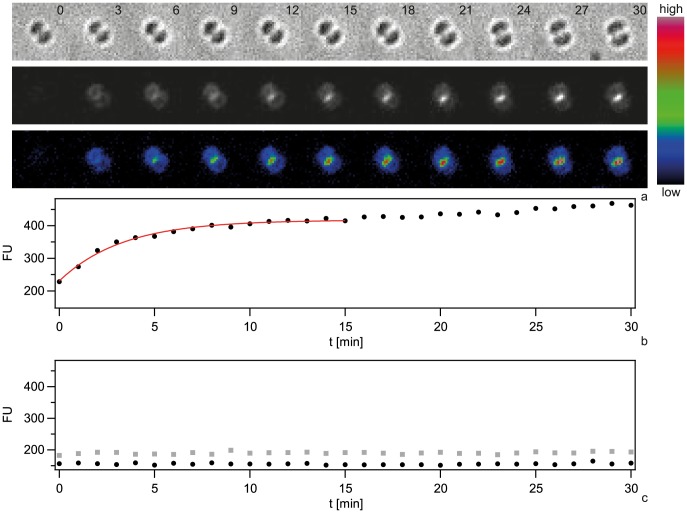
Dynamics of focus formation (*ΔpilV*) with 300 bp fragments of Cy3-DNA. a) Time lapse of binding and import of Cy3-DNA. Upper line: brightfield image, middle line: fluorescence image, lower line: pseudocolored intensity image. Δt = 3 min. b) Black circles: Fluorescence intensities of cells shown in a. Red line: Exponential fit. c) Fluorescence intensities of individual *ΔpilQ ΔpilV* (black circles) and *ΔpilT ΔpilV* (grey boxes).

We next investigated the temporal dynamics of Cy3-DNA during DNA uptake by monitoring single *ΔpilV* cells during incubation with 300 bp Cy3-DNA in real-time. The fluorescence intensity per cell showed a saturating kinetics with *FU(t) = FU_max_(1−exp(−t/τ))* with τ = (4.5±0.6) min (Fig. 4 a, b, Movie S1). FU_max_ is a measure for the total amount of Cy3-DNA that can be imported into the periplasm. Cy3-DNA tended to accumulate at the septa of diplococci. To test whether the kinetics characterized binding or import of Cy3-DNA, we repeated the experiment in *ΔpilTΔpilV* and *ΔpilQΔpilV* backgrounds. During 30 min, we did not detect an increase in fluorescence intensity (Fig. 4 c), demonstrating that Cy3-DNA was imported with a characteristic time of 4.5 min in the *ΔpilV* strain.

In conclusion, the periplasm saturates with short Cy3-DNA fragments within minutes.

### ComE quantitatively increases the total amount of Cy3-DNA imported into the periplasm

**Figure 5:**
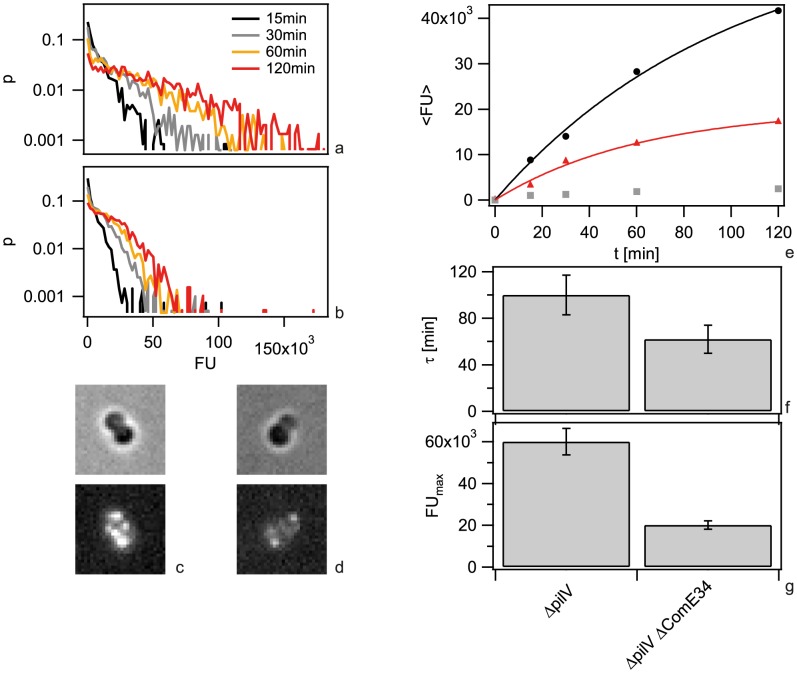
Dynamics of 3-DNA import depends on ComE. a, b) Probability distribution of the total fluorescence intensity of individual a) *ΔpilV* and b) *ΔcomE_34_ ΔpilV* as a function of time. c, d) Typical fluorescence images after 1 h for c) *ΔpilV* and d) *ΔcomE_34_ ΔpilV*. e) Average fluorescence intensity per cell as a function of time with 3 kbp Cy3-DNA for *ΔpilV* (black circles), *ΔcomE_34_ ΔpilV* (red triangles), *ΔcomE_1234_ ΔpilV* (grey squares). Full lines: fits to exponential function. f) Saturation value for average fluorescence intensity per cell obtained from fits in e). g) Characteristic times obtained from fits in e). (N>1500 for each condition).

It has been shown previously that ComE is necessary for DNA uptake into a DNAse-resistant state and transformation [16]. We investigated whether ComE acted by increasing the carrying capacity of the periplasm or by speeding up DNA import. To this end, we generated isogenic backgrounds varying in *comE* copy number. Since the amount of imported Cy3-DNA was similar for the backgrounds with four and three copies and for one or no *comE* copies (Fig. S7), we concentrated on strains with two (*ΔcomE_34_*) versus no (*ΔcomE_1234_*) alleles in the following. In a first set of experiments, we incubated *ΔpilV* cells with 3 kbp DNA for variable amounts of time before treating them with DNase (Fig. 5 a,b). Comparing the patterns formed in *ΔpilV* and *ΔcomE_34_ΔpilV* at various time points did not reveal a striking difference. For example, at 1 h both mutants showed multiple foci (Fig. 5 c, d). The kinetics could be well described by a single exponential function *FU(t) = FU_max_(1−exp(−t/τ))* (Fig. 5e). In the *ΔcomE_34_ΔpilV* strain, that carries two copies of the *comE* gene, the capacity was decreased by a factor of ∼3 as compared to the *ΔpilV* strain (Fig. 5g). The complete *comE* deletion strain *ΔcomE_1234_ΔpilV* showed a decrease of fluorescence intensity by a factor of ∼24. This reduction is similar to the reduction in a *pilQ* deletion strain in agreement with ComE being necessary for DNA uptake. We note, however, that residual fluorescence was observed in some cells. The characteristic time to saturation was τ = (100±17) min in *ΔpilV*. If ComE would enhance the speed of DNA import, then we would expect that reduction of the ComE concentration leads to an increase in the characteristic time. Instead, we observed a decrease to τ = (62±12) min in *ΔcomE_34_ΔpilV* (Fig. 5f).

**Figure 6:**
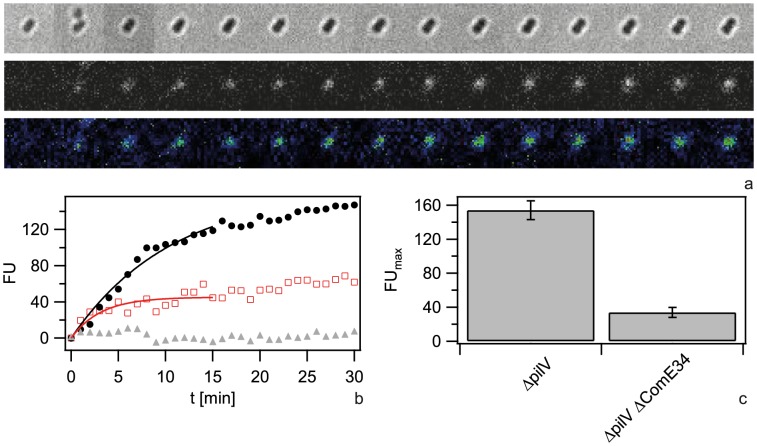
Dynamics of 300-DNA import depends on ComE. a) Time lapse of binding and import by *ΔcomE_34_ ΔpilV*. Upper line: brightfield image, middle line: fluorescence image, lower line: pseudocolored intensity image. Δt = 2 min. b) Single cell kinetics for *ΔpilV* (black circles), *ΔcomE_34_ ΔpilV* (red triangles), *ΔcomE_1234_ ΔpilV* (grey squares). Full lines: fits to exponential function. c) Average saturation value for fits as in b). (N>6 for each condition).

Since we found that ComE increased the carrying capacity of the periplasm, we tested whether ComE was necessary for importing very short DNA fragments of 100 nm. Since Cy3-DNA import showed saturation within minutes, we quantified single cell fluorescence in real-time during incubation with 300 bp Cy3-DNA (Fig. 6). We found that Cy3-DNA import of short fragments was dependent on ComE. A full *comE* null mutant did not show any increase in fluorescence, indicating that ComE was essential for Cy3-DNA import. The saturating fluorescence intensity *F_max_* was strongly decreased in the *ΔcomE_34_* strain, confirming that ComE quantitatively controls the carrying capacity of the periplasm.

### ComE-mCherry relocalizes to DNA-foci

**Figure 7:**
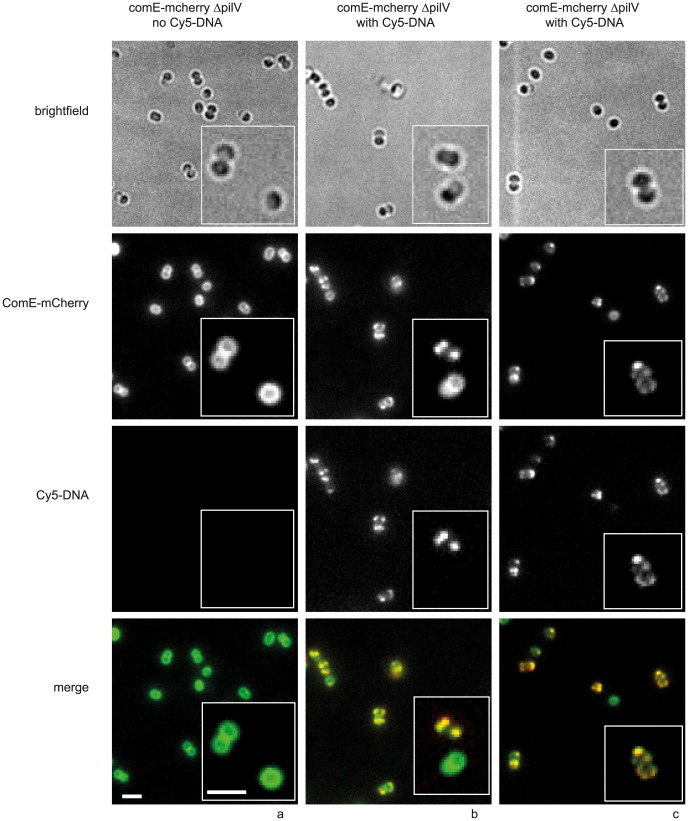
ComE-mCherry colocalizes with the spotty pattern of Cy5-DNA. *comE*-*mcherry* cells were incubated with a) no DNA, b, c) 10 kbp Cy5-DNA fragments for 15 min and visualized. Scale bars: 2.5 µm, insets: 2× magnification.

As Cy3-DNA was not homogeneously distributed within the periplasm (Fig. 1), we investigated whether the periplasmic DNA-binding protein ComE co-localized with DNA. To this end, we generated a strain in which one of the *comE* genes was fused to an *mcherry* ORF. In the absence of transforming DNA, ComE-mCherry showed a mostly ring-like distribution, indicating that it was homogeneously distributed within the periplasm (Fig. S8a). Some cells showed pronounced foci which were most often located at the septa between the cocci of diplococci. To test whether these foci arouse from DNA that was present due to lysed cells, we incubated cells with DNase for 30 min and subsequently let the bacteria grow for three generations in liquid culture. Upon this treatment, the foci disappeared almost completely revealing homogeneous distribution of ComE-mCherry in the periplasm (Fig. S8b, Fig. 7a).

In the next step, we incubated the *comE-mcherry ΔpilV* cells with 3 kbp Cy5-DNA for 15 min. The distribution of mCherry-fluorescence became spotty, often revealing distinct foci (Fig. 7b, c), reminiscent of the fluorescence pattern generated by imported Cy5-DNA. The patterns of Cy5-DNA and ComE-mCherry were highly correlated, indicating co-localization between ComE and imported DNA (Fig. 7 b, c). Most cells that had little or no Cy5-DNA signal retained their ring-like ComE-mCherry fluorescence (e.g. Fig. 7 b inset).

In summary, ComE-mCherry is homogenously distributed in the periplasm in the absence of transforming DNA. Imported DNA forms foci in the periplasm and co-localization of ComE indicates that the periplasmic DNA interacts with ComE.

### Spatio-temporal dynamics of Cy3-DNA foci and ComE-mCherry in the periplasm

**Figure 8:**
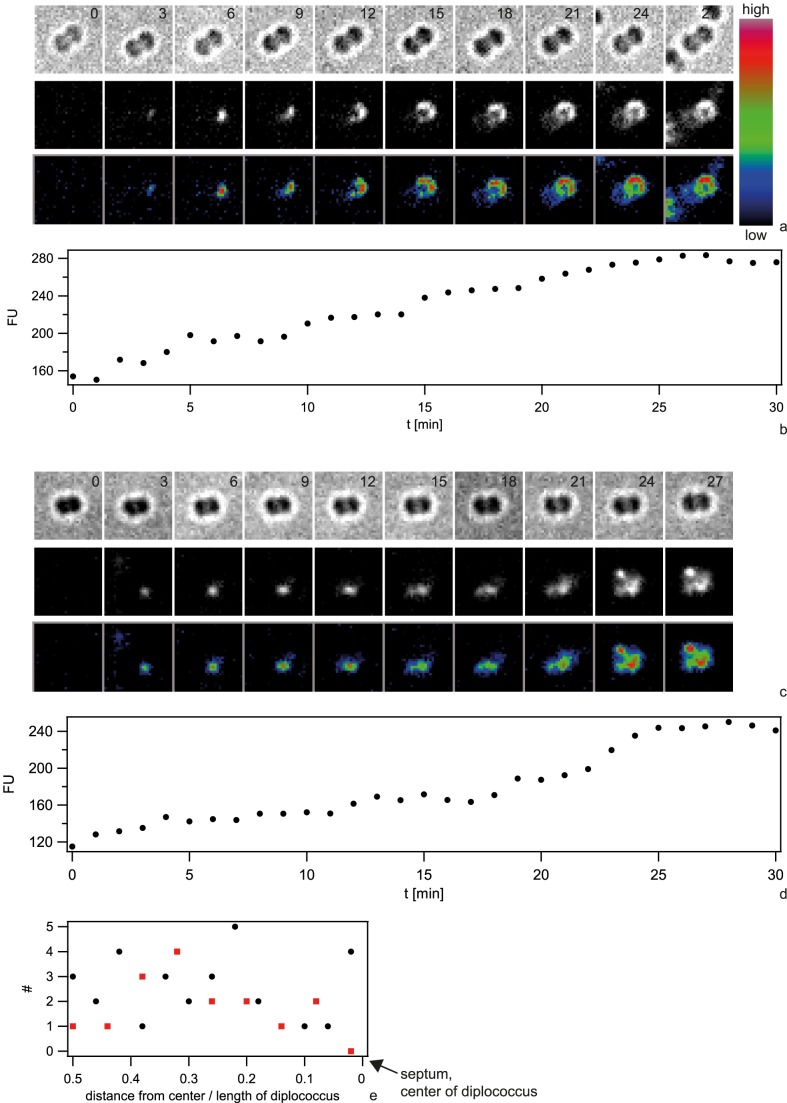
Dynamics of focus formation (*ΔpilV*) with 10 kbp fragments of Cy3-DNA. a, c) Examples for time lapse of binding and import. Upper line: brightfield image, middle line: fluorescence image, lower line: pseudocolored intensity image. *Δ*t = 3 min b, d) Fluorescence intensities of cells shown in a, c). e) Distribution of initial location of Cy3-DNA foci normalized to length of diplococcus. Black circles: Cy3-DNA, red squares: YOYO-DNA.

We investigated the spatio-temporal dynamics of the foci/spotty pattern of Cy3-DNA in the periplasm. To determine the initial location of Cy3-DNA after import, we acquired a time lapse of 10 kbp Cy3-DNA import. Individual 6 kbp Cy3-fragments are clearly visible (Fig. S4). Indeed, we observed the successive appearance of well-defined fluorescent spots correlated with a step-wise increase of fluorescence intensity (Fig. 8 a–d). These foci showed little movement over a time scale of 60 min (Movies S2, S3). To investigate whether there was a preferred location of DNA import (as reported for *B. subtilis*), we projected the location of initial occurrence to the major axis of a diplococcus and normalized this distance to the cell length (1 min time resolution). Only diplococci were analysed in order to find the location relative to the septum. We found no preferred location of DNA uptake (Fig. 8e). Using DNA that was labeled with the reversibly intercalating dye YOYO, we repeated the experiment. Again, no preferential location of YOYO-DNA foci was observed (Fig. 8e).

**Figure 9:**
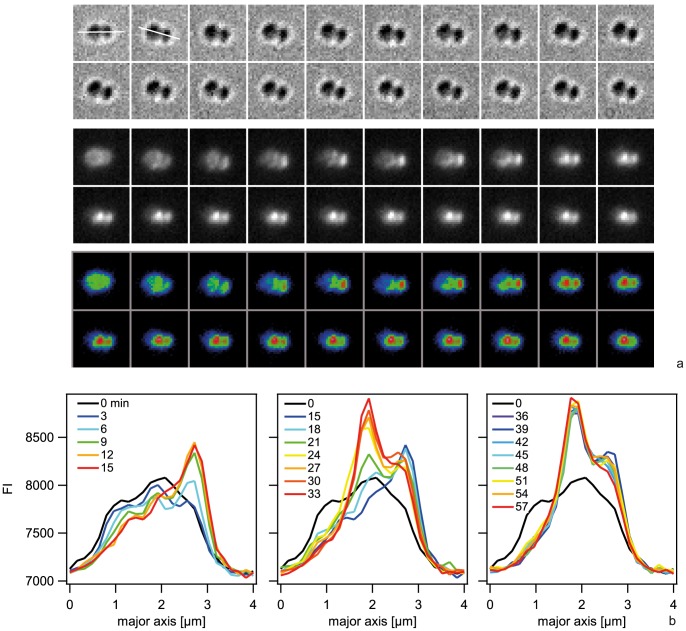
Spatio-temporal dynamics of ComE-mCherry upon addition of 10 kbp transforming DNA. a) Time-lapse of ComE-mCherry dynamics. Upper line: brightfield image, middle line: fluorescence image, lower line: pseudocolored intensity image. The line denotes the major axis. *Δ*t = 3 min. b) Intensity profiles through the major axis at different time points.

Next, we investigated the spatio-temporal dynamics of ComE-mCherry during exposure to unlabeled 10 kbp transforming DNA. These experiments were performed at 37°C and therefore the image quality was not comparable to Fig. 7. Initially, the fluorescence intensity was homogeneous or ring-like (Fig. 9, Movie S4). Spontaneously, the fluorescence accumulated to form a focus whereas the fluorescence in the reminder of the cell decayed. The intensity of the focus increased for ∼10 min and was then stable. At 18 min a second focus appeared again taking ∼15 min to reach its maximum intensity. This second focus was stable for another 30 min. Monococci also formed stable multiple foci (Movie S5). This experiment indicates that the formation of stable foci is not caused by fluorescence labeling of DNA.

**Figure 10:**
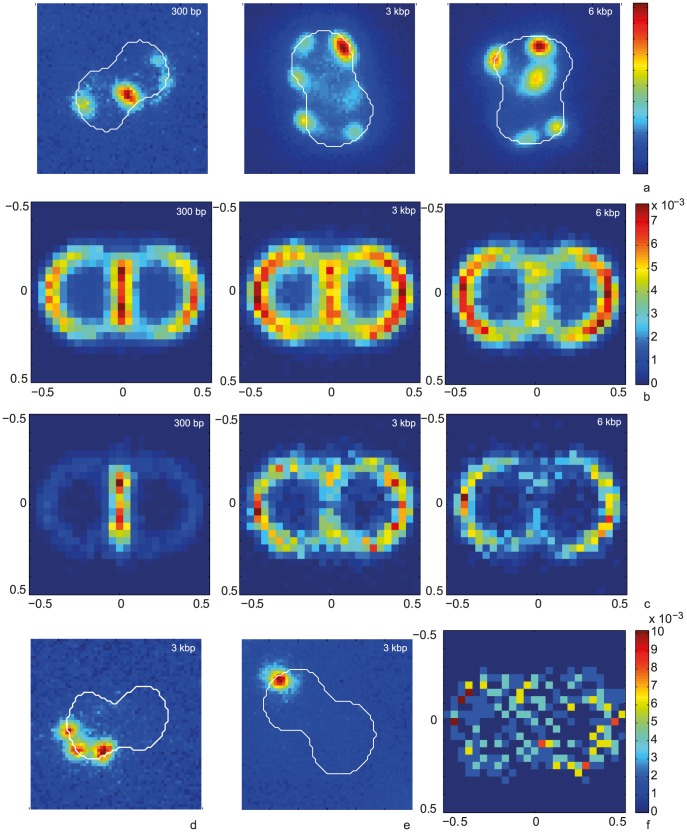
Spatial distribution of Cy3-DNA foci after 15 min of exposure to transforming Cy3-DNA in *ΔpilV* and wt. a) Typical examples of Cy3-DNA single cell fluorescence for different fragment lengths (*ΔpilV*). The white line denotes the cell contour as obtained from the bright field image. b) Two-dimensional distribution of focus location (*ΔpilV*). The color encodes the probability for finding a focus within the 2D bin. (N>18000 for each condition). c) Two-dimensional distribution 10% brightest foci (*ΔpilV*). d, e) Typical examples of Cy3-DNA single cell fluorescence for different fragment lengths (wt). f) Two-dimensional distribution of focus location (wt) (N = 309).

To characterize the distribution of focus location, we optimized the imaging conditions using a camera with higher pixel density and we imaged at room temperature. *ΔpilV* cells were incubated with Cy3-DNA at 37°C for 15 min, treated with DNase and subsequently imaged. For different Cy3-DNA fragments, we found foci around the cell contour (Fig. 10a). We wrote an algorithm that detected diplococci, defined the cell contour, aligned them, and normalized the cell sizes. With this algorithm, the two-dimensional probability distribution of focus location was plotted (Fig. 10b). We found that foci were located all around the cell contour with a slight bias towards the −0.5/0.5 position for long fragments. To assess whether particularly bright foci had a different distribution, we plotted the distribution of the 10% brightest foci (Fig. 10c). Very clearly, the 300 bp fragments accumulated at the septa between the cocci, in agreement with Fig. 4a. Finally, we assessed the distribution of 3 kbp Cy3-DNA in wt cells. Again we found that foci formed (Fig. 10 d,e). Although the total number of foci was low, we found that foci were distributed all around the cell contour.

**Figure 11:**
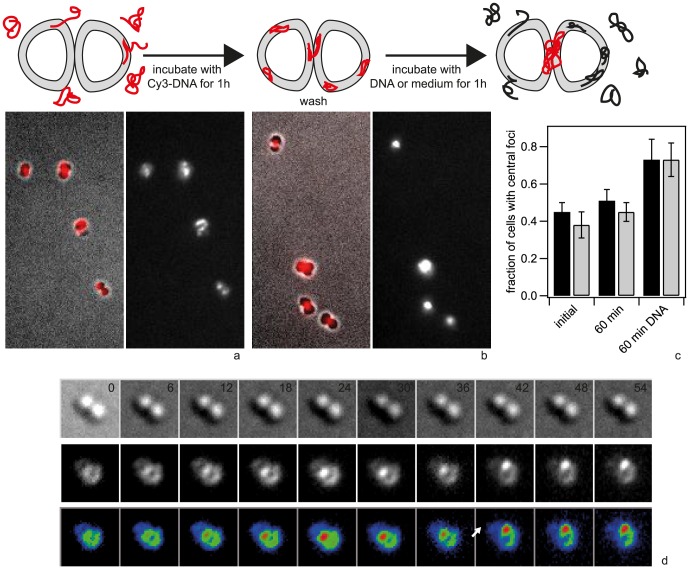
Redistribution of 3-DNA. a) Typical example after 1 h incubation with Cy3-DNA and subsequent washing. Left: superposition of brightfield and fluorescence image, right: fluorescence image. b) Typical example of the same sample after washing and subsequent incubation with unlabeled DNA for another 1 h. left: superposition of brightfield and fluorescence image, right: fluorescence image. c) Fraction of cells with foci at septum directly after 60 min incubation with Cy3-DNA, after 60 min incubation with medium, and after 60 min incubation with unlabeled DNA. black: *ΔpilV*, grey: *ΔcomA ΔpilV*. (N>1000 for each condition) d) Time-lapse of central focus formation and movement. Upper line: brightfield image, middle line: fluorescence image, lower line: pseudocolored intensity image. The arrow denotes the timepoint where the focus moves. *Δ*t = 6 min.

To test whether there is re-organization of long Cy3-DNA over longer periods of time, we incubated cells for 1 h with 3 kbp Cy3-DNA fragments, washed them and subsequently incubated with unlabeled DNA. We found that the fraction of cells showing central foci increased strongly with time (Fig. 11a, b, c). This behavior was independent of ComA. Time lapse microscopy revealed formation of central foci (Fig.11d, Movie S6) and movement of central foci (Fig. 11d). When cells were incubated for one hour without unlabeled DNA, the pattern did not change significantly (Fig. 11b, c).

In summary, our data strongly suggests the following spatio-temporal dynamics. Transforming DNA is imported at random locations. DNA fragments form foci with associated ComE that are recruited at the septa. The time scale of recruitment depends on DNA length.

## Discussion

### Putative role of the periplasmic protein ComE

We have shown that ComE-mCherry is homogeneously distributed within the periplasm in the absence of transforming DNA, i.e. after DNase treatment and prolonged incubation in liquid culture. When harvested without DNase treatment, a fraction of cells showed foci that were mostly at the septa between cocci. This finding strongly suggests that gonococci accumulate DNA presumably released by lysis of siblings or by secretion in the periplasm while growing in microcolonies. Most importantly, upon addition of Cy5-DNA, ComE-mCherry localized to the Cy5-DNA foci. These experiments demonstrate that ComE is free to move within the periplasm and is captured by periplasmic DNA. While this manuscript was in revision, it has been reported that the homolog ComEA-mCherry diffuses rapidly in the periplasm of *V. cholerae* and co-localizes with YOYO-DNA [36]. Together with experiments showing that expression of *N. gonorrhoeae-comE* can restore functionality in a *V. cholerae-comEA* deletion mutation, ComE-dependent DNA-import into the periplasm can be considered a general mechanism for Gram-negative competent species. Our data further show that association of ComE-mCherry with Cy5-DNA is not a transient phenomenon but that ComE remains associated with periplasmic DNA. Interestingly, the homologue of ComE in *B. subtilis*, ComEA, is the only known competence protein that does not localize to the cell pole [28]. It has been suggested, that ComEA molecules sequester DNA all around the cell shuffling the DNA to the site of DNA uptake where the rate-limiting step of DNA import occurs [29]. Similarly, in gonococci ComE is likely to compact DNA and to increase the capacity of the periplasmic reservoir.

Indeed, we found that ComE quantitatively governed the carrying capacity of the periplasm. Since the length of the imported DNA fragments did not influence the carrying capacity, we conclude that the ComE concentration is a limiting factor for the amount of DNA that the periplasm can hold. With a height of ∼20 nm [37] and a cell radius of ∼0.4 µm, the periplasm has a volume of ∼3·10^−5^ µm^3^. Considering a 10 kbp DNA fragment and assuming no excluded volume interactions, the volume of the statistical coil with a radius of gyration of 235 nm assumes ∼5·10^−2^ µm^3^, indicating that energy must be spent for packaging the DNA. To assess whether the function of ComE is to package the DNA, we incubated gonococci with DNA fragments of the order of the Kuhn segment length of 100 nm (300 bp). The Kuhn segment length is a measure for the stiffness of the polymer, in other words it describes over which length the polymer can be bent by Brownian motion. We found, however, that ComE was essential for the import of 300 bp fragments into the periplasm. Mechanistically, we propose that during DNA import, DNA binds weakly to the machine that pulls the DNA through the outer membrane. To render the import irreversible ComE binds to the incoming DNA and hinders its backward movement, generating a translocation ratchet [26]. Additionally, ComE might compact the DNA beyond its Kuhn segment length of 100 nm.

### Spatio-temporal dynamics of DNA import and maintenance within the periplasm

Using DNA fragments with a length of 10 kbp, we found that Cy3-DNA foci occurred at random locations around the cell contour. As the time resolution of our time-lapse acquisition was 1 min, this location most likely coincides with the location of import through the outer membrane. The 10 kbp DNA fragments did not show strong mobility over the period of 30 min. The stochastic distribution of DNA import complexes correlates with the distribution of T4P in gonococci [38], supporting the idea that DNA directly interacts with components of the T4P system. However, we did not observe T4P-like structures coated with Cy3-DNA during real-time experiments. Therefore, we cannot draw conclusions about the potential role of T4P fibers directly in DNA uptake. This result strongly suggests that multiple DNA import complexes exist and are distributed around the cell contour. In *B. subtilis* and *H. pylori*, DNA import was found to be localized either to the cell pole or to the septum [28] [16] [24]. In *B. subtilis*, one or two DNA import complexes or accumulations of the latter were stable even when the cell wall was dissolved resulting in cellular deformation to a round morphology [29]. Mostly single T4P pili were found in *V. cholerae* that were randomly located around the cell contour [39]. Here, we found that DNA was imported from multiple sites in a single bacterium. We speculate that bacteria that use T4P uniquely for DNA import may form a single pilus, whereas *N. gonorrhoeae* is a peritrichously piliated bacterium in agreement with multiple DNA import sites reported here.

Interestingly, when cells were incubated with 3 kbp Cy3-DNA and subsequently with unlabeled DNA, Cy3-DNA foci tended to move to septa of diplococci. These dynamics were observed only in the presence of unlabeled transforming DNA. Similar central accumulations were observed with 300 bp Cy3-DNA after several minutes. One possible explanation would be that newly incoming DNA pushes the previously imported DNA away from the sites of DNA uptake. The local accumulation of periplasmic DNA is somewhat reminiscent of DNA-filled membrane blebs that have been termed “transformasome” in *Haemophilus influenzae* [40], [41]. In TEM-images these membrane-blebs were found at a 10× increased frequency in competent versus non-competent *H. influenzae*. These sites were proposed to be the sites of DNA-import.

The lipoprotein Tetrapac (Tpc) is essential for transformation and deletion of this putative murein hydrolase shows a severe defect in cell separation generating tetrapacs instead of diplococci [18]. It is reasonable to assume that Tpc acts at the septum by hydrolyzing the cell wall. Periplasmic DNA would then be trapped at the septum location where Tpc degrades the cell wall. Furthermore, DNA was shown to bind to FtsE which is believed to be involved in cell division through interaction with FtsZ which localizes to the septum [42]. Very recently, it has been shown that the pneumococcal EndA nuclease localizes to the midcell upon induction of competence [32]. Interestingly, fluorescently labeled Cy3-DNA also localized to the midcell of competent pneumococci, suggesting that active uptake occurs at this location. Furthermore, they found that midcell localization was independent of *comEC* expression in agreement with our finding that midcell localization was independent of *comA*. It is tempting to speculate that recruitment of DNA to the site of future cell division is a general property of competent cocci although it is currently unclear whether this accumulation is linked to DNA transport through the inner membrane.

### Power and limitations of the Cy3-DNA approach

Here we have introduced a novel approach for studying the spatio-temporal dynamics of DNA import in Gram-negative species at the single cell level. So far, DNA import has been mostly studied at the population level. We have verified that the static properties agree with previous reports using radioactively labeled DNA, including the effect of the outer membrane pore [7], the inner membrane channel [17], the minor pilins [7], [11], and the periplasmic proteins [16]. We have previously quantified the effect of Cy3-labels on the speed of DNA-import in *H. pylori* and found that the import speed was decreased by a factor of ∼2 [24]. Considering that DNA import depends on the pore formed by PilQ and that its inner opening is wide enough to allow translocation of the type IV pilus, we do not expect that the speed of DNA import is strongly influenced by the Cy3-label. One clear limitation of our approach is the fact that Cy3-DNA is not transported into the cytoplasm and as a consequence, transport through the inner membrane cannot be characterized. However, we performed saturation experiments using unlabeled DNA, to verify that accumulation of DNA in the periplasm is not caused by Cy3-labeling. When cells were pre-incubated with unlabeled genomic DNA or with PCR-fragments for 1 h, the fluorescence signal obtained after subsequent incubation with Cy3-DNA was strongly reduced. This experiment verifies that massing of DNA within the periplasm occurs with unlabeled DNA and is not caused by Cy3. Moreover, ComE-mCherry foci formed after treatment with unlabeled transforming DNA and were stable for up to 30 min. This experiment further supports the claim that stable periplasmic DNA foci are not caused by Cy3-labeling. Previous experiments with *H. pylori* showed that YOYO-DNA was imported into the periplasm [24]. Here, we found YOYO-DNA binding to the gonococcal surface, but the signal was lost upon DNase treatment (data not shown). This finding might be caused either by rapid formation of ssDNA in the periplasm, by expulsion of the dye from dsDNA due to DNA compaction in the periplasm, or by quenching.

Most experiments were performed in a *pilV* deletion background to facilitate imaging. This strain has been reported to show a strong increase in the level of both DUS-specific DNA uptake and in the rate of transformation [11]. Thus, transport through the inner membrane is not the only bottleneck for transformation and the accumulation of DNA in the periplasm is not likely to be caused by *pilV* deletion. Our experiments show that the gonococcal periplasm is saturable with DNA in a ComE-dependent manner, but it remains unclear, whether the wt reaches saturation. However, within biofilms, gonococci are surrounded by a large amount of genomic DNA and a high abundance of DUS [43]. Being continuously exposed to large amounts of DUS containing DNA and potentially dividing at a much lower rate, it is conceivable, that saturation is achieved by the wild type in biofilms. In our experiments, the Cy3-DNA fragments contained only a single DUS, however the deletion of *pilV* increases DUS-dependent binding and uptake, so using the *pilV* mutant in our experimental setup is most likely mechanistically not very different to the wildtype situation in biofilms. Concerning the molecular mechanism of DNA import, the *pilV* strain is not different to that seen in wt backgrounds as DNA import depends on *pilE*, *pilT*, DUS, and on assembled pili [11]. In wt gonocoocci, the distribution of DNA-foci was similar to the *ΔpilV* background and imported DNA was stable as well. All things considered, we propose that Cy3-labeling of DNA is a useful tool for studying the spatio-temporal dynamics of DNA import into the periplasm.

### Conclusion

Our results support the following spatio-temporal dynamics of DNA uptake. DNA transport through the outer membrane is powered by DNA uptake complexes that are randomly distributed over the cellular contour. The periplasmic DNA-binding protein ComE is homogeneously distributed in the periplasm in the absence of DNA. Upon contact with periplasmic DNA, it relocalizes to foci formed by DNA. The carrying capacity of the gonococcal periplasm for DNA depends on ComE in a gene-dosage-dependent fashion. Nanomanipulation experiments will be necessary to investigate whether ComE has an additional role in directly driving DNA-import through the outer membrane. When external DNA is present, the periplasmic DNA is relocated to septa of diplococci. It is tempting to speculate that midcell/septum location of transport through the cytoplasmic membrane is conserved between Gram-positive and Gram-negative cocci. It will be interesting to assess whether the location of inner membrane transport is at the septum.

## Materials and Methods

### Bacterial strains and growth conditions

*N. gonorrrhoeae* (Table S1) was grown overnight at 37°C and 5% CO_2_ on agar plates containing gonococcal base agar (10 g/l Bacto agar (BD Biosciences, Bedford, MA, USA), 5 g/l NaCl (Roth, Darmstadt, Germany), 4 g/l K_2_HPO_4_ (Roth), 1 g/l KH_2_PO_4_ (Roth), 15 g/l Proteose Peptone No. 3 (BD), 0.5 g/l soluble starch (Sigma-Aldrich, St. Louis, MO, USA)) and the following supplements: 1 g/l D-Glucose (Roth), 0.1 g/l L-glutamine (Roth), 0.289 g/l L-cysteine-HCL×H_2_0 (Roth), 1 mg/l thiamine pyrophosphate (Sigma-Aldrich), 0.2 mg/l Fe(NO_3_)_3_ (Sigma-Aldrich), 0.03 mg/l thiamine HCl (Roth), 0.13 mg/l 4-aminobenzoic acid (Sigma-Aldrich), 2.5 mg/l β-nicotinamide adenine dinucleotide (Roth) and 0.1 mg/l vitamin B_12_ (Sigma-Aldrich). Before each experiment gonococcal colonies were resuspended in GC-medium.

### Construction of mutant strains

*ΔpilQ ΔpilV*, *ΔpilT ΔpilV*, and *ΔcomA ΔpilV* were constructed by transforming genomic DNA from existing deletion mutants in the N400 background into GV1 (*ΔpilV*) [44] (Table S1). The genomic DNA was isolated from GQ21 [45], GT17 [46] and *ΔcomA* (derived by transformation of N400 with the *ΔcomA* allele originally detailed in Facius and Meyer 1993 [19]). The constructions of the *ΔcomE* strains are described in the Supplementary Methods (Text S1). Antibiotics and IPTG were used at the following concentrations: 50 µg/ml kanamycin (*Roth*), 50 µg/ml apramycin (*Sigma-Aldrich*), 2.5 µg/ml erythromycin (*Sigma-Aldrich*), 10 µg/ml chloramphenicol (*Roth*), 40 µg/ml spectinomycin (*Sigma-Aldrich*), 2 µg/ml tetracyclin (*Roth*), 1 mM IPTG (*Roth*).

### DNA-labeling

The covalent attachment of Cy3 and Cy5 dyes to DNA was achieved with the help of the *Label IT Nucleic Acid Labeling Kits* (Mirus). According to the manufacturer, the Label IT reagent is bound by a reactive alkylating group to any reactive heteroatom of the DNA without altering the structure of the nucleic acid. The labeling reagent, labeling buffer and 5 µg of DNA were mixed in Milli-Q-H_2_O to a total volume of 50 µl according to the manufacturer's protocol with a 1∶1 (v/w) ratio of Label IT reagent to DNA. The incubation time at 37°C was elongated to 2 h to improve the labeling density. The samples were purified subsequently by using the provided microspin columns. For comparative quantifications, only labeled DNA from the same labelling reaction was used. YOYO-1-iodide (*Invitrogen*) is a dimeric cyanine with a highly specific affinity to bind to DNA. Due to its structure, it can easily intercalate between single base pairs, modifying the DNA less than covalently attached dyes. We used a dye∶bp ratio of 1∶50. For this, 1 µg of DNA in 10 µl Milli-Q-H_2_O was mixed with 0.3 µl of a freshly diluted 0.1 mM YOYO solution in Milli-Q-H_2_O.

### DNA uptake assays

Several bacterial colonies of 16 h–20 h old cultures grown on GC-agar were resuspended with a 10 µl inoculation syringe in 100 µl DNA-uptake-medium (GC-medium supplemented with Isovitalex and 7 mM MgCl_2_) to an OD_600_ of 0.1. Cy3- or YOYO-labeled PCR-fragments were added to the cell suspension to a final concentration of 1 ng/µl. The cells were incubated with DNA at 37°C with 5% CO_2_ and subsequently treated with 10 U DNAse I (recombinant, *Fermentas*) for further 15 min at 37°C. 50 µl of this dilution were applied to cover slides for microscopic analysis. For each comparative experiment, the different strains or conditions were characterized on the same day using the same stock of labeled DNA. Each condition was characterized independently on at least three different days.

### Microscopy and quantitative analysis of single cell fluorescence

All fluorescence quantification and real-time experiments were conducted at an inverted microscope (Eclipse TE2000 *Nikon*) in epi-fluorescence mode at 37°. A 120 W metal halogenide fluorescence lamp (X-Cite, *EXFO*) served as illumination source. Images were taken with an EMCCD camera (Cascade II:512, *Photometrics*). The 100× oil immersion CFI Plan Fluor objective (NA 1.3, *Nikon*) was used in all applications. Day to day variations in the brightness of the fluorescence lamp were detected by using the test beads on the *Focal Check Fluorescence Microscope Test Slide #3* (*Invitrogen*). The intensities from day to day stayed relatively stable with no more deviations than ±10%. A correction factor to this reference intensity was calculated for every day and was applied to the respective data sets. The analysis is described in the Supplementary Methods (Text S1) and Fig. S2.

For localization experiments an inverted microscope (TI-E *Nikon*) was used at room temperature. A 120 W metal halogenide fluorescence lamp (Intensilight *Nikon*) served as illumination source. Images were taken with a CCD camera (Orca 2.3 *Hamamatsu*) that had a high pixel density for Cy3-DNA focus distribution. For ComE-mCherry/Cy5-DNA co-localization, an EMCCD camera (IXON X3897 *Andor*) was used.

### Real-time quantification of DNA import

Cells selected for piliation were grown on a GC agar plate for <20 h, harvested, resuspended in DNA-uptake-medium and adjusted to OD_600_ = 0.01–0.02. 150 µl of the suspension were applied to a polystyrene-coated coverslip inside a microscopy chamber and incubated 10 min on the microscope stage. Subsequently, 1.4 µg of 300 bp Cy3-DNA diluted in 100 µl DNA-uptake-medium was added and DNA uptake was recorded for 60 min. To control for DNA bound to the exterior of the cells, 10 U/ml of DNaseI (*Fermentas*) was added and fluorescence intensity was recorded for another 15 min.

## Supporting Information

Figure S1 **The minor pilins PilV and ComP differentially influence the amount of imported Cy3-DNA.** Upper line: merged images of brightfield and fluorescence, lower line: fluorescence images. Gonococci were incubated with Cy3-DNA for 30 min and subsequently treated with DNase. a) wt with enhanced contrast, b) wt with contrast adjusted to c, d. c) *P_pilE_comP*, d) *ΔpilV*.
(EPS)

Figure S2 **Quantification of single cell fluorescence.** a) Cells are automatically detected in the bright-field channel and accepted (green squares) or rejected (red squares) depending on shape or distance to neighbors. b) Regions of interest (ROIs) of size 30×30 pixel are taken about the center of each bacterium for single cell analysis. Left: brightfield, center: fluorescence, right: merge. c) Blue bars: Histogram of intensity of individual pixels in ROI (30×30). The local background is determined by a gauss fit over the intensity distribution inside each ROI (red line). After background subtraction the sum over all pixel values inside a ROI gives the value for single cell fluorescence.
(EPS)

Figure S3 **Comparison between monococci and diplococci.** Quantification of total single cell fluorescence after incubation with 3 kbp Cy3-DNA for 30 min.
(EPS)

Figure S4 **Quantification of imported Cy3-DNA.** a) Typical image of single 6 kbp Cy3-DNA fragments immobilized on a cationic lipid membrane [33]. Blue squares: ROI for fluorescence signal of single DNA fragment, yellow squares: ROI for background subtraction for adjacent DNA fragment. b) Grey: Distribution of fluorescence intensities of single 6 kpb Cy3-DNA. Full line: Gaussian fit with a center of mass of (6010±120) FU. c) Grey: Distribution of single cell fluorescence after incubation of the *ΔpilV* strain with 6 kbp Cy3-DNA from the same batch for 1 h. Full line: Gaussian fit with a center of mass of (39621±586) FU.
(EPS)

Figure S5 **Duplex PCR of imported DNA.** Imported DNA fragments were amplified at varying time periods after DNase treatment for a) *ΔpilV* b) wt.
(EPS)

Figure S6 **Turnover of Cy3-DNA in the periplasm.** Gonococci were incubated for 1 h with 3 kbp Cy3-DNA (black), washed, and subsequently treated with no DNA (grey) or unlabeled DNA (red) for 1 h. Cumulative histograms of fluorescence intensities of individual cells for a) *ΔpilV*, b) *ΔpilV ΔcomA*.
(EPS)

Figure S7 **Fluorescence distribution of individual cells with varying** ***comE*** **expression.** a) After 1 h incubation with 3 kbp Cy3-DNA. b) *ΔpilV* c) *ΔpilV ΔcomE_34_* d) *ΔpilV ΔcomE_1234_* after varying periods of incubation with 3 kbp Cy3-DNA.
(EPS)

Figure S8 **Distribution of ComE-mCherry in the absence of transforming DNA.** a) Example of mCherry-Fluorescence and intensity plots through the fluorescence images along the two axes indicated in red. b) When cells were scratched from the agar plate and imaged directly, some cells showed foci which were most often located at the septum between the cocci. When cells were treated with DNase for 30 min and subsequently grown for three generations, the foci disappeared almost completely.
(EPS)

Movie S1 **Example for dynamics of focus formation (** ***ΔpilV*** **) with 0.3 kbp fragments of Cy3-DNA.** Left: brightfield, right: fluorescence with *Δ*t = 1 min (Fig. 7a).
(AVI)

Movie S2 **Example for dynamics of focus formation (** ***ΔpilV*** **) with 10 kbp fragments of Cy3-DNA.** Left: brightfield, right: fluorescence with *Δ*t = 1 min (Fig. 6a).
(AVI)

Movie S3 **Example for dynamics of focus formation (** ***ΔpilV*** **) with 10 kbp fragments of Cy3-DNA.** Left: brightfield, right: fluorescence with *Δ*t = 1 min (Fig. 6b).
(AVI)

Movie S4 **Spatio-temporal dynamics of ComE-mCherry** during exposure to unlabeled DNA (diplococcus). Left: brightfield, right: fluorescence with *Δ*t = 1 min. (corresponding to Fig. 9).
(AVI)

Movie S5 **Spatio-temporal dynamics of ComE-mCherry** during exposure to unlabeled DNA (monococcus). Left: brightfield, right: fluorescence with *Δ*t = 1 min.
(AVI)

Movie S6 **Redistribution of Cy3-DNA foci.** Left: brightfield, right: fluorescence with *Δ*t = 3 min. (corresponding to Fig. 11d).
(AVI)

Text S1 **Supplementary Methods.** Description experimental methods and data analysis.
(PDF)
