# Supplementary material for: Concerted Spatio-Temporal Dynamics of Imported DNA and ComE DNA Uptake Protein during Gonococcal Transformation
Source: PLoS Pathog. 2014 Apr 24;10(4):e1004043. doi: 10.1371/journal.ppat.1004043 (PMC3999279; doi:10.1371/journal.ppat.1004043)
Supplement: Text S1 — Supplementary Methods. Description experimental methods and data analysis. (PDF) [file ppat.1004043.s015.pdf]

Supplementary material for

Concerted spatio-temporal dynamics of imported DNA and ComE DNA uptake protein during gonococcal transformation

Heike Gangel[1]*, Christof Hepp[1]*, Stephanie Müller[1]*, Enno R. Oldewurtel[1], Finn Erik Aas[2], Michael Koomey[2], Berenike Maier[1]

Supplementary Methods

Construction of deletion strains.

Single comE deletions. Genomic DNA of a complete knockout of all four *comE* copies in MS11 has been kindly provided by Ines Chen [1]. The genomic DNA was transformed into N400 and the cells were plated separately on the four different antibiotic markers: Erythromycin for *comE2*, chloramphenicol for *comE3* and kanamycin for *comE4* (note: the markers were distributed slightly different than in the publication cited above). Genomic DNA was isolated from *comE2*, *comE3* and *comE4* in the N400 background strains for further constructions of knockouts in GV1. Unfortunately, the insertion of the Omega-fragment, which carries the spectinomycin resistance cassette, in *comE1* resulted in a negative polar effect on the downstream *comP* [1], although *comP* is apparently not organized in an operon with *comE1*. The reason for the knockdown effect remains elusive, but possibly the large inserted Omega fragment and the distance between the stop-codon of the spectinomycin resistance gene to the following gene region downstream of *comE1* caused this strong polar effect. Hence, a non-polar knockout of *comE1* has been constructed in this work. For this, the apramycin resistance gene from pUC1813Apra was amplified using primers NG51 and NG52 and has been inserted directly into the start and stop codons of *comE1* using overlap extension PCRs with the amplified up and downstream regions of *comE1*. The GTG start codon of apramycin has been replaced by the original *comE1* start codon ATG in this step. All regulatory units like the promoter regions or possible Shine Dalgarno sequences up or downstream of *comE1* should be preserved by this procedure. As the transformation of PCR-fragments, although containing the DUS, did not work with the *comE1*-knockout construct, the upstream region of *comE1* was amplified for the overlap extension PCR with primers NG53-2 and NG54, inserting a *Eco*RI restriction site into the fragment (marked bold in NG53-2). In the downstream region, a *Bam*HI restriction site (marked bold in NG56-2) was inserted by the amplification with primers NG55 and NG56-2. The restriction sites were necessary for the incorporation of the *comE1::Apra* fragment into the transformation vector

pUP6. After fusing the *comE1* upstream and downstream PCR fragments with the apramycin resistance cassette using the outer primers NG53-2 and NG56-2, both the PCR-fragment and the backbone vector pUP6 were double digested with *Eco*RI and *Bam*HI. Subsequently, the PCR fragment was ligated into the linearized plasmid after dephosphorylation of the 5'- sticky endings of pUP6. The construct was transformed into GV1 and cells were selected on apramycin.

Gradual comE deletions. For the construction of multiple *comE* knockouts, the genomic DNA of these single knockouts in GV1 was isolated. Then, a successive transformation of genomic DNA produced following gradual knockouts: *comE4/3* by transforming genomic DNA from *comE3* into *comE4*; *comE4/E3/E2* by transforming *comE2* into *comE4/E3*; and finally, *comE4/E3/E2/E1* by transforming *comE1* into *comE4/E3/E2*.

All single and gradual *comE* knockout strains were controlled via PCR for the correct genotype in all four *comE* loci after transformation and selection on the respective antibiotic markers. The upstream regions of the *comE* loci are homologue to each other up to approximately 6000bp (according to the sequence from strain FA1090), so the same primer, NG2, was always used as upstream primer for all *comE* loci. For this reason, special care was taken that the downstream primers bind very specifically to the respective downstream *comE* loci. In particular, *comE1* is the only locus with no homologue region to the other three *comE*-loci directly after the stop codon, whereas *comE2*, *comE3* and *comE4* share the same downstream sequence up to approximately 150bp, *comE2* and *comE4* even up to 350bp. Thus, all downstream primers were designed at least 600bp downstream of the respective *comE* stop codon. These primers were NG23 for *comE1*, NG24-2 for *comE2*, NG25 for *comE3* and NG26 for *comE4*.

comE-mcherry fusion. ComE4 was selected to be replaced with a C-terminal fusion construct of *comE* and *mcherry*, connected by a $(PS)_4$ spacer. For this purpose a PCR construct was designed, comprising the *comE-mcherry* fusion, an antibiotic resistance cassette and the 5' and 3' flanking regions of the *comE4* locus required for homologous recombination. The *comE*-ORF and its 5' flanking region was amplified by PCR using primers KHpam1 and KHpam2imp. The coding sequence for mCherry was amplified from *pGCC4-mCherry* by primers KHpam3 and KHpam4, introducing the spacer at the 5'end. The kanamycin resistance cassette was amplified from *pUP6* by KHpam5 and KHpam6, introducing a Shine-Dalgarno sequence at its 5'end. The 3' region of ComE4 was amplified by primers KHpam7 and KHpam8. All fragments were joined together by fusion PCR to the construct, which was directly transformed into *N.gonorrhoeae* GV1. To verify the correct insertion of the construct, colony PCR of single clones was performed using primers NG2 and NG26. The PCR product was used as a template for sequencing of the recombinant – *comE-mcherry* construct.

Fragments for DNA import. Fragments for DNA uptake were amplified from λ-DNA (Roche) by PCR. The forward primer was either containing or lagging a DUS (NGCH001 and NGCH002). Fragment length was defined by the reverse primers, yielding fragments of 300 bp, 1kbp, 6 kbp, 10 kbp by use of primers NGCH003, NGCH005, NGCH008, NGCH009 respectively.

Quantification of Cy3-DNA import at the single cell level.

Cells were analyzed by routines written in MATLAB. Bacteria were automatically detected in the bright-field channel and accepted or rejected depending on shape and distance to the nearest neighbors. Thus positions of individual single mono- or diplococci were found. Optionally the automatic selection was further refined manually (Fig S1 a, b). Each cell was assigned a 30x30 pixels large region of interest (ROI). The pixel intensities I inside a ROI were fitted with a Gaussian function, $g(I) = a \cdot exp(-(I - I_{back})^2/b^2)$, to determine the local background. After background subtraction by shifting the distribution by $-I_{back}$ the sum over the pixel intensities gives a measure for the total single cell fluorescence (Fig S1 c). This routine allowed to quickly analyze a large number of cells and gain information over the total fluorescence of each cell, irrespective of a strongly localized or weakly diffusive distribution.

Characterization of saturation.

Several 16-18 h old colonies were resuspended in 100 µl DNA-uptake-medium to an OD_{600} of 0.1. A 3 kbp unlabeled PCR fragment with a single DUS or isolated genomic DNA from an MS11 strain were added to a final concentration of 1 ng/µl and the samples were incubated at 37°C and 5% CO_2 for 60 min. A control sample was prepared and incubated in parallel without any additions. All samples were washed twice by centrifuging (1 min, 2000 g) and replacing the supernatant with fresh, pre-warmed DNA-uptake-medium to remove unlabeled DNA from the sample. 3 kbp Cy3-DNA was added to a final concentration of 1 ng/µl and the samples were incubated at 37°C and 5% CO_2 for 30 min and treated with DNase (0.2 U/µl, Thermo Scientific) for 15 min. Microscopy samples were prepared from 50 µl of the cell suspension and kept on ice until imaging to prevent turnover in the meantime.

Duplex PCR for investigating degradation of DNA in the periplasm.

Cells grown on GC-agar for 16-10 h were resuspended in 500 µl of DNA-uptake-medium to an OD_{600} of 0.1, incubated with 1 ng/µl 10 kbp PCR fragment containing a single DUS at 37°C for 60 min and treated with DNAseI (10 U/ml, Fermentas) for 15 min. To test for degradation over a larger timescale DNAseI incubation time was extended. To remove DNAse along with residual DNA, the samples were vortexed for 3 min and washed in DNA-uptake-medium several times. The pellets were resuspended in MilliQ-H_2O and incubated at 98°C for 5 min, vortexed shortly and pelleted at 13000 rpm for 5 min. The supernatant was stored at -20°C or immediately used for the PCR reaction. PCR was carried out using DreamTaq MasterMix (Fermentas). Genomic DNA was amplified using primers NG2 and NG23, while PCR fragment was amplified using primers NGCH002 and B260, yielding a 3 kbp PCR product. The ratio between genomic and fragment primers in the PCR sample was 1:15. In the GV1 background, the annealing temperature was increased to 65°C to suppress the formation of unspecific PCR artifacts.

Characterization of Cy3-DNA turnover.

Several 16-18 h old colonies were resuspended in 100 µl DNA-uptake-medium to an OD_{600} of 0.1. 3 kbp Cy3-DNA with a single DUS was added to a final concentration of 1 ng/µl and the sample was incubated at 37°C and 5% CO_2 for 60 min. The cells were washed twice by centrifuging (1 min, 2000 g) and replacing the supernatant with fresh, pre-warmed DNA-uptake-medium. The control samples were imaged immediately after this step. The other samples were incubated further for 60 min with or without 10 ng/µl of the unlabeled 3 kbp fragment containing a single DUS before imaging. Microscopy samples were prepared from 50 µl of the cell suspension and kept on ice until imaging.

Automatic detection of foci.

The position of foci with respect to cell center and orientation were found. MATLAB routines were used to find the contour shape of single cells in the brightfield channel and the DIPimage toolbox for MATLAB allowed to analyse the shape. The information of contour, intensity distribution in the brightfield channel captured before the fluorescent channel and comparison with another image taken in the brightfield channel after fluorescence capture were used to accept only cells well adhered and flatly lying in the right focus plane, which match the shape characteristics of diplococcus cells. The orientation of the contour was defined to be the angle perpendicular to the axis of the minimal Feret diameter of the contour. Knowing this angle and the centre of the contour allowed to rotate every cell into the same orientation. Finally, the length of the pixel mask bounded by the contour was used to normalize all diplococcus cells into a common coordinate system.

For foci detection the image of the fluorescent channel was smoothed using a Gaussian convolution kernel. Subsequently, local peaks with intensity values significantly above the background were identified to be foci. The contour length and centre previously found in the brightfield channel allowed to transfer the foci position into the same common coordinate system. The intensity values of the peaks after background subtraction were recorded to distinguish between dim and very bright foci. It should be noted that foci coordinates were only normalised by the length of the cell. However, the analysed diplococci can have varying degrees of pinching between the cocci. Hence foci right at the end of the cell are mostly mapped into the same location in the common coordinate system, while foci right at the neck of the cell are mapped into a slightly larger area depending on the degree of pinching of the respective cell.

Supplementary References

1. Chen I, Gotschlich EC (2001) ComE, a competence protein from Neisseria gonorrhoeae with DNA-binding activity. J Bacteriol 183: 3160-3168.

2. Tonjum T, Freitag NE, Namork E, Koomey M (1995) Identification and characterization of pilG, a highly conserved pilus-assembly gene in pathogenic Neisseria. Molecular microbiology 16: 451-464.
3. Freitag NE, Seifert HS, Koomey M (1995) Characterization of the pilF-pilD pilus-assembly locus of Neisseria gonorrhoeae. Molecular microbiology 16: 575-586.
4. Aas FE, Wolfgang M, Frye S, Dunham S, Lovold C, et al. (2002) Competence for natural transformation in Neisseria gonorrhoeae: components of DNA binding and uptake linked to type IV pilus expression. Mol Microbiol 46: 749-760.
5. Winther-Larsen HC, Hegge FT, Wolfgang M, Hayes SF, van Putten JP, et al. (2001) Neisseria gonorrhoeae PilV, a type IV pilus-associated protein essential to human epithelial cell adherence. Proceedings of the National Academy of Sciences of the United States of America 98: 15276-15281.
6. Wolfgang M, van Putten JP, Hayes SF, Dorward D, Koomey M (2000) Components and dynamics of fiber formation define a ubiquitous biogenesis pathway for bacterial pili. Embo J 19: 6408-6418.
7. Aas FE, Winther-Larsen HC, Wolfgang M, Frye S, Lovold C, et al. (2007) Substitutions in the N-terminal alpha helical spine of Neisseria gonorrhoeae pilin affect Type IV pilus assembly, dynamics and associated functions. Molecular microbiology 63: 69-85.
8. Facius D, Meyer TF (1993) A novel determinant (comA) essential for natural transformation competence in Neisseria gonorrhoeae and the effect of a comA defect on pilin variation. Mol Microbiol 10: 699-712.
9. Maier B, Radler JO (2000) DNA on fluid membranes: A model polymer in two dimensions. Macromolecules 33: 7185-7194.

Supplementary tables

Table S1

Strain	Relevant genotype	Source/Reference
N400	*recA6ind(tetM)*	[2] [3]
GP117	*recA6ind(tetM)*<br>*iga::P_{pilE}comP erm^R*	[4]
GV1	*recA6ind(tetM) pilVfs (G-1)*	[5]
ΔpilQΔpilV	*pilQ::m-Tn3cm*<br>*recA6ind(tetM) pilVfs*	This work, [6]
ΔpilTΔpilV	*pilT::m-Tn3cm*<br>*recA6ind(tetM) pilVfs*	This work, [7]
ΔcomAΔpilV	*comA::TnMax1*<br>*recA6ind(tetM) pilVfs*	This work, [8]
ΔcomE1ΔpilV	*comE1::Apra*<br>*recA6ind(tetM) pilVfs*	This work
ΔcomE2ΔpilV	*comE2::Erm*<br>*recA6ind(tetM) pilVfs*	This work*, [1]
ΔcomE3ΔpilV	*comE3::Clm*<br>*recA6ind(tetM) pilVfs*	This work*, [1]
ΔcomE4ΔpilV	*comE4::Kan*<br>*recA6ind(tetM) pilVfs*	This work*, [1]
ΔcomE34ΔpilV	*comE4::Kan*<br>*comE3::Clm*<br>*recA6ind(tetM) pilVfs*	This work
ΔcomE1234ΔpilV	*comE4::Kan,*<br>*comE3::Clm,*<br>*comE2::Erm,*<br>*comE1::Apra*<br>*recA6ind(tetM) pilVfs*	This work
comE-mcherry ΔpilV	*pilE4-mcherry Kan*<br>*recA6ind(tetM) pilVfs*	This work

** constructed with genomic DNA kindly donated by Ines Chen*

Table S2

Primers	
NG51	5’-CAATACGAATGGCGAAAAGCCGAG-3’
NG52	5’-TCATGAGCTCAGCCAATCGACTG-3’
NG53-2	5’GC**GAATTC**ATGCCGTCTGAAGCCCGTGAGGCTTGACTCTAT-3’
NG54	5’CTCGGCTTTTCGCCATTCGTATTGCATGGTTTTTCCTTTAAGGGTTGCAAAC-3’
NG55	5’-CAGTCGATTGGCTGAGCTCATGAAGTGGGAAATATGCATACTGCTGAATG-3’
NG56-2	5’-GC**GGATCC**ATGCCGTCTGAACCATCAAGGCGGAAAACCGCAC-3’
NG2	5’-ATGGCGGGACGGCATCTGTAC-3’
NG23	5’-CAGCAGGACAAGGTTCGCGATG-3’
NG24-2	5’-GCAAAAGGAGCTTGCCTTGAACAGC-3’
NG25	5’-CCGATCGAACAATACCTGAACCGTC-3’
NGCH001	5'-ATGCCGTCTGAACGGTTTAAGGCGTTTCCGTTCTTC-3'
NGCH002	5'-CGGTTTAAGGCGTTTCCGTTCTTC-3'
NGCH003	5'-GGCATACCATTTTATGACGGCGG-3'
NGCH005	5'-CATCACCATCCGTCGGCAACC-3'
NGCH008	5'-CCGTTTTTTCGTCTCGTCGCTGG-3'
NGCH009	5'-CGACGAATTTTCGCCGCCCC-3'
KHpam1	5'-GGATCCATGCCGTCTGAACGTCGCAAGATGCGG-3'
KHpam2imp	5‘TGAAGGCGATGGTGACGGAGATGGTTTTTTAACCGCAGGCAGCACCGGTTTGGCGGG-3'
KHpam3	5'-CCATCTCCGTCACCATCGCCTTCAATGGTGAGCAAGGGCGAGG-3'
KHpam4	5'-TTATTCCTCCTAGTTAGTCACTTGTACAGCTCGTCCATGCCG-3'
KHpam5	5'CTGTACAAGTGACTAACTAGGAGGAATAAATGATTGAACAAGATGGATTGCTCGCAG-3'
KHpam6	5'-CCCCTTCCTTTACAGGTTCCCCTATCAGAAGAACTCGTCAAGAAGGCGATAG-3'
KHpam7	5'-TAGGGGAACCTGTAAAGGAAGGGGCATCGGCTGCCGCCGGC-3'
KHpam8	5'-GAATTCTTGCCTTCCACCCTTCG-3'
